# Breastfeeding in Cystic Fibrosis: A Systematic Review on Prevalence and Potential Benefits

**DOI:** 10.3390/nu13093263

**Published:** 2021-09-18

**Authors:** Carla Colombo, Gianfranco Alicandro, Valeria Daccò, Alessandra Consales, Fabio Mosca, Carlo Agostoni, Maria Lorella Giannì

**Affiliations:** 1 Fondazione IRCCS Ca’ Granda Ospedale Maggiore Policlinico, Cystic Fibrosis Center, 20122 Milan, Italy; gianfranco.alicandro@unimi.it (G.A.); valeria.dacco@policlinico.mi.it (V.D.); 2 Department of Pathophysiology and Transplantation, Università degli Studi di Milano, 20122 Milan, Italy; 3 Fondazione IRCCS Ca’ Granda Ospedale Maggiore Policlinico, NICU, 20122 Milan, Italy; alessandra.consales@unimi.it (A.C.); fabio.mosca@unimi.it (F.M.); maria.gianni@unimi.it (M.L.G.); 4 Department of Clinical Sciences and Community Health, Università degli Studi di Milano, 20122 Milan, Italy; carlo.agostoni@unimi.it; 5 Fondazione IRCCS Ca’ Granda Ospedale Maggiore Policlinico, Pediatric Unit, 20122 Milan, Italy

**Keywords:** cystic fibrosis, breastfeeding, infant feeding, growth, infections, pulmonary outcomes

## Abstract

Breastfeeding (BF) is considered the normative standard of feeding for all infants. However, the impact of BF in patients with cystic fibrosis (CF) is not completely defined. Therefore, we conducted a systematic review to evaluate BF prevalence in the CF population and its impact on anthropometric and pulmonary outcomes. We searched MEDLINE, Embase and the Cochrane Library for original articles published in English up to 4 December 2020 that report the prevalence of BF and/or any measure of association between BF and anthropometric or pulmonary outcomes. Nine observational studies were identified (six retrospective cohort studies, one prospective cohort study, one survey and one case–control study within a retrospective cohort). The BF rate in CF patients is lower than that of the healthy population (approximately 50–60% of infants were breastfed at any time). The benefits in anthropometric outcomes of BF for >2 months in this at-risk population are unclear. A few relatively small studies suggest a potential benefit of BF in reducing lung infections, although data are inconsistent. The currently available data are insufficient to draw definite conclusions on the benefits of exclusive BF in anthropometric and pulmonary outcomes in CF. Clinical trials evaluating well-defined BF promotion interventions are needed.

## 1. Introduction

Cystic fibrosis (CF) is a life-shortening autosomal recessive genetic condition affecting more than 90,000 people worldwide [1]. It is caused by mutations in the CF transmembrane conductance regulator (CFTR) gene, which alters the physiological sodium and chloride transport, resulting in viscous secretions. CF is a multisystem disease affecting the respiratory, gastrointestinal and reproductive tracts, and the sweat glands. 

For many years, breastfeeding (BF) for infants with CF was discouraged, mainly due to concerns regarding reports of hypoproteinemia, hyponatremia and vitamin D and E deficiency. Due to the associated limitations in the absorption of fats and fat-soluble vitamins, BF was considered inadequate, in terms of energy supply, protein and sodium content, to meet the increased requirements of infants with CF, particularly for those with pancreatic insufficiency (PI) and meconium ileus (MI) [2,3]. However, these events are unlikely to be related to BF per se and, in the modern era of CF nutritional care, can be easily prevented with close monitoring of nutritional status, adequate sodium and vitamin supplementation, and pancreatic enzyme replacement therapy (PERT). 

Starting from the 1990s, the increasing evidence of several benefits of BF in healthy infants led to a change in perspective, with 77% of CF centers recommending BF alone or in any combination with hydrolyzed formula and PERT, if required. However, it was only in 2002 that the CF Foundation Consensus approved BF as the recommended primary source of nutrition in the first year of life for patients with CF [4]. Accordingly, the 2016 ESPEN-ESPGHAN-ECFS guidelines [5] recommended exclusive BF for newly diagnosed infants with CF, highlighting the need for specific advice regarding PERT, salt supplements and nutritional intake. However, the optimal duration of exclusive BF was not indicated, leaving the WHO general recommendations [6] as the main indication. 

Therefore, collecting evidence on the potential benefits of BF for infants with CF would be important to give evidence-based recommendations to parents in order to provide infants with CF with the best nutritional support from early life. 

To this aim, we conducted a systematic review on BF prevalence in CF and its relationship with anthropometric and pulmonary outcomes.

## 2. Materials and Methods

We searched MEDLINE, Embase and the Cochrane Library databases for original articles published in English up to 4 December 2020 that report the prevalence of BF and/or any measure of association between BF and anthropometric or pulmonary outcomes. Citations were exported from the databases and then imported to Rayyan for title and abstract screening [7]. Weight, length/height and BMI (expressed as raw values or z-scores for age and sex), as well as weight-for-length z-scores, were considered as anthropometric outcomes, while the number of pulmonary exacerbations, IV antibiotic treatments, hospitalizations, lung infections, radiological scores of lung disease and forced expiratory volume in one second (FEV_1_) were considered among pulmonary outcomes.

We included articles reporting randomized clinical trials and observational studies, while reviews, opinion papers, conference proceedings and studies where the analysis was not based on individual data were excluded. The following search terms were used for the search: “cystic fibrosis” AND (“breastfeeding” OR “breastfed”). Titles and abstracts were screened, and the full texts of the eligible articles were obtained. The references included in the full text of the eligible articles were manually searched for studies that could have been missed.

From the included articles, we extracted the following data: year of publication, country where the study was conducted, study design, number of enrolled patients, prevalence and duration of BF, groups compared, measured outcomes and corresponding results.

Since we did not find any randomized clinical trial related to the objectives of our systematic review, we used the Newcastle–Ottawa Scale to assess the risk of bias in each cohort or case–control study [8]. The risk of bias for studies reporting results from surveys was evaluated trough the response rate.

The study findings were reported according to the Preferred Reporting Items for Systematic Reviews and Meta-Analyses (PRISMA) guidelines [9].

## 3. Results

### 3.1. Selected Articles

**Figure 1:**
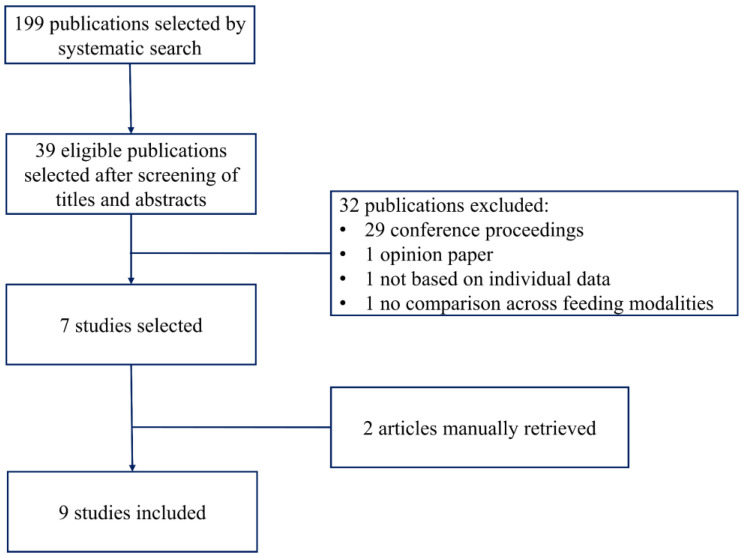
Flow diagram of the studies selection.

Figure 1 shows the flow diagram of the studies included in this review. 

The systematic search yielded 199 unique items; 39 were considered eligible after screening of the title and abstract. Thirty-two publications were excluded: 29 were published as conference proceedings, one article was an opinion paper [10], one article was a survey among CF center directors that did not report individual data [11] and one article [12] reported preintervention and postintervention values of growth parameters after a project to improve BF in CF, but it did not show a comparison between breastfed and formula-fed infants. Thus, seven studies were identified by the systematic search [13,14,15,16,17,18,19], and two studies were identified by the manual search [20,21].

Table 1 gives a summary overview of the nine selected articles: six retrospective cohort studies, one prospective cohort study, one survey and one case–control study within a retrospective cohort.

The risk of bias for the cohort and case–control studies was generally low, although some comparability issues emerged (Table 2). In fact, the majority of the studies contained no or only partial control for important confounders, including PI, MI and socioeconomic status. With regard to the only survey [13] included in this systematic review, the response rate was very low (27%), with only 868 questionnaires returned out of 3200 sent, leaving doubts about the representativeness of the survey population.

### 3.2. Prevalence and Duration of Breastfeeding

All nine identified studies reported the prevalence of BF in CF (Table 3).

The prevalence of BF was approximately 50–60%. The BF rate was found to be higher among patients with PS than among patients with PI and MI. Overall, BF prevalence was consistently lower compared to that of the general population, and this was also the case for exclusive BF rate. However, the figures provided by the included studies are difficult to compare since they were collected at different time points. 

### 3.3. Breastfeeding and Anthropometric Outcomes

Six studies investigated the relationship between BF and anthropometric outcomes in CF (Table 4).

The association between BF and anthropometric outcomes failed to reach statistical significance in the majority of the studies. The multi-center retrospective cohort study by Jadin et al. showed a rapid decline in weight-for-age z-scores from birth to six months of age only in children who had been exclusively breastfed for more than two months [15]. In contrast, Leung et al. found a positive association between exclusive BF and weight-for-age z-scores at 3 months of age, but this result was not confirmed at 6 and 12 months of age [17]. A positive association between BF and weight-for-age z-scores at 12 and 24 months of age was also found in a small subgroup (N = 16) of patients with MI [20].

### 3.4. Breastfeeding and Pulmonary Outcomes

Seven studies investigated the relationship between BF and pulmonary outcomes in CF (Table 5). 

All studies evaluated the association between BF and lung infections, while only two studies also considered respiratory function indicators [13,14], one of which showed a positive association [14]. Colombo et al. reported a lower number of infections over the first three years of life among infants who had been breastfed for more than four months as compared to those who had been formula-fed or breastfed for a shorter period [14]. Parker et al. found lower IV antibiotic use over two years before the enrollment in their survey in patients who had been exclusively breastfed for more than six months as compared to formula-fed patients [13]. Among the three studies investigating P. *aeruginosa* acquisition or colonization [15,16,18], one study found an inverse association [15], and two studies found null associations [16,18], although one of them [18] reported a trend toward a later acquisition among exclusively or partially breastfed infants. 

However, only one study evaluated the association between BF and a radiological score, showing higher scores among infants who had been breastfed for at least 2 months as compared to those breastfed for a shorter period [15].

One study on CF patients with MI evaluated a composite outcome including P. *aeruginosa* infection and faltering growth, reporting a higher risk of negative outcome among infants who were never breastfed as compared to breastfed infants, but the result was not statistically significant [19].

## 4. Discussion

The studies identified for the present systematic review are consistent in reporting a lower prevalence of BF among infants with CF than among healthy infants. Based on these studies, the impact of BF on anthropometric and pulmonary outcomes remains uncertain because data are based on relatively small studies that are largely heterogeneous in terms of design and findings. 

BF prevalence is much lower than that reported in healthy infants, and it is initiated on average in 50% of newborn infants with CF. Data on BF duration are even scarcer, but seem to indicate that it is also shorter, particularly in patients with PI and MI.

The decision of mothers to discontinue BF is based on several factors, including personal perceptions of the adequacy of breast milk (BM), poor social and family support, and difficulties for women in reconciling work with child’s care [22]. All these barriers play a more important role for mothers of children with CF, who have to face many challenges soon after diagnosis, including the psychological stress associated with the newly diagnosed disease, the frequent failure to thrive of their infants and the multiple therapies prescribed to prevent the progression of the disease [12]. Moreover, the management of CF therapy in breastfed children requires further arrangements to ensure proper PERT and adequate supplementations of sodium, iron and vitamins; this may lead both the parents and the physician to decide on the early discontinuation of BF. Notably, iron supplementation can paradoxically limit the highly efficient iron absorption from BM, by “blocking” lactoferrin [23,24,25]. 

On the other hand, initiatives to promote and support BF among mothers of children with CF have recently shown promising results in reducing the discontinuation of BF. In a recent study by Miller et al. [12], an International Board-Certified Lactation Consultant (IBCLC) was incorporated into the initial CF-diagnosis visit in order to support mothers who were already BF. After the intervention, 94% of mothers (16 out of 17 mothers) continued BF vs. 57% of the pre-intervention group (8 out of 14 mothers). Duration of BF and exclusive BF was increased, although not significantly, to an average of 7.7 and 4.5 months, respectively, compared to the 6.4 and 3.6 months in the pre-intervention group.

With regard to the relationship among BF, anthropometric and pulmonary outcomes, the available data are limited, based on a relatively small number of patients, and heterogeneous in terms of measured outcomes, comparisons and results. Overall, the results on anthropometric outcomes are controversial, while there is some evidence that BF may reduce or delay lung infections in CF. 

Most studies did not find significant differences in anthropometric outcomes across different feeding modalities. Nevertheless, two studies suggested some benefits of BF for patients with MI, possibly impacting the later expression of the individual growth potential [19,20]. Accordingly, one study documented a positive association between BF and growth rate in 16 infants [20] and the other, based on 85 patients, reported a reduced risk of faltering growth and/or chronic P. *aeruginosa* infection at 1 year of age (evaluated as a composite outcome) among breastfed compared to never breastfed infants with CF [19]. It is well known that human milk contains high concentrations of antimicrobial proteins, including lactoferrin, lysozyme, lactadherin and HMO, and growth factors, such as transforming growth factor beta, epidermal growth factor and insulin-like growth factor, which may improve the gut microbiome in these patients [26].

Since only a few relatively small studies suggest a potential benefit of BF in reducing lung infections, the relationship between BF and pulmonary outcomes remains unclear. Most studies did not consider potential confounding variables (e.g., higher socio-economic status and maternal level of education in the BF group), and the majority had a retrospective design with possible recall bias. 

When interpreting the results of the studies reported in this systematic review, some important issues related to the different periods in which they were carried out should be considered. First, the aggressive market campaigns in the 1970s and 1980s strongly influenced maternal feeding choices, and at that time, formula feeding was more frequent than in recent years. Second, the composition of formula milk has been improved over time to mimic BM, both in terms of nutritional and functional effects. Finally, the small amount of included studies, the short follow-up for some of them, and the high heterogeneity in the timing of the measurements, comparisons and outcomes should also be considered. This last issue prevented us from providing a quantitative summary measure of the association between BF and CF outcomes through a meta-analysis.

## 5. Conclusions

In conclusion, our systematic review indicates that BF can be recommended in CF, since infants who are breastfed even for a prolonged period of time do not show a compromised growth, provided that they are monitored in specialized centers according to the Standards of Care [27]. MI does not seem to represent a contraindication to BF, even if only a few small studies considered the effect of BF in this subgroup of infants, who are at increased risk of growth faltering. 

Large randomized clinical trials evaluating interventions of BF promotion with well-defined feeding strategies are needed to draw definitive conclusions on the positive effects of BF on CF outcomes.

## Figures and Tables

**Table 1:** Summary overview of the selected studies.

First Author (Year of Publication), Country [Reference]	Study Design	Enrolled Patients
Holliday et al. (1991), Australia [20]	Single-center retrospective cohort study	65 CF infants without MI and 16 infants with MI
Parker EM et al. (2004), USA [13]	Multicenter survey	768 CF patients
Colombo et al. (2007), Italy [14]	Single-center retrospective cohort study	146 patients (aged 5–18 years) seen at the CF center between September 2003 and April 2004
Jadin et al. (2011), USA [15]	Multicenter retrospective cohort study	103 CF infants born between 1994 and 2006 who were diagnosed through NBS
Rosenfeld et al. (2012), USA [16]	Multicenter retrospective cohort study	264 CF patients aged < 2 years with no isolation of *P. aeruginosa* from any respiratory culture prior to enrollment
Leung et al. (2017), USA [17]	Multicenter prospective cohort study	231 CF infants younger than 3.5 months diagnosed by NBS
Munck et al. (2018), France [18]	Multicenter prospective cohort study	105 infants with no history of MI recruited between April 2010 and September 2011 after the first visit for confirmatory CF diagnosis after NBS
Padoan et al. (2019), Italy [19]	Multicenter case–control study within a retrospective cohort study	85 CF infants with MI born between 2009 and 2016 and diagnosed by symptoms
Fitzgerald et al. (2020), Ireland [21]	Multicenter retrospective cohort study	186 CF infants (77 born between July 2008 and June 2011 clinically diagnosed and 109 born between July 2011 and June 2016 diagnosed through NBS)

CF: cystic fibrosis; MI: meconium ileus; NBS: newborn screening.

**Table 2:** Risk of bias for the included case–control and cohort studies according to the Newcastle–Ottawa scale.

First Author (Year of Publication) [Reference]	Study Design	Selection	Comparability	Exposure ^a^	Outcome ^a^	Score (Maximum: 9)
Holliday et al. (1991) [20]	Cohort	4	0	NA	3	7
Colombo et al. (2007) [14]	Cohort	4	2	NA	3	9
Jadin et al. (2011) [15]	Cohort	4	1	NA	3	8
Rosenfeld et al. (2012) [16]	Cohort	4	0	NA	3	7
Leung et al. (2017) [17]	Cohort	4	0	NA	3	7
Munck et al. (2018) [18]	Cohort	4	1	NA	3	8
Padoan et al. (2019) [19]	Case/control within a cohort	4	0	3	NA	7
Fitzgerald et al. (2020) [21]	Cohort	4	2	NA	3	9

NA: not applicable. ^a^ According to the Newcastle–Ottawa scale, points for exposure are scored only for case–control studies and points for outcome only for cohort studies. Higher scores indicate a lower risk of bias.

**Table 3:** Summary overview of the studies reporting on prevalence of breastfeeding in cystic fibrosis.

First Author (Year of Publication) [Reference]	Prevalence of Breastfeeding
Holliday et al. (1991) [20]	Patients without MI: 63.1% BF at least 3 m, 46.2% exBF. Patients with MI: 43.8% BF at least 3 m.
Parker EM et al. (2004) [13]	49% BF at any time, 18% exBF.
Colombo et al. (2007) [14]	62% BF at any time, 23% BF > 4 m. BF rate at any time higher among PS patients (74%) than PI patients (57%).
Jadin et al. (2011) [15]	51% BF at any time (26% exBF < 1 m, 10.7% ExBF = 1 m and 13.6% exBF ≥ 2 m). BF rates at any time higher among PS patients (66%) than PI patients without MI (48%) and patients with MI (54%).
Rosenfeld et al. (2012) [16]	Ever BF: 52.3%.
Leung et al. (2017), USA [17]	BF at 3 m of age: 43.3%, 25% exBF.
Munck et al. (2018) [18]	At initial visit the rates of exclusively or partial BF were 47% among PI patients and 20% among PS patients. Corresponding figures at 6 m: 20% among PI patients and 28% among PS patients. 47.6% of patients received exclusive BF during the first 3 m of life.
Padoan et al. (2019) [19]	Ever BF in the first 3 m of life: 54%.
Fitzgerald et al. (2020) [21]	Ever BF: 51.4% among the NBS group and 54.2% among the clinically diagnosed group.

BF: breastfed; CF: cystic fibrosis; exBF: exclusively breastfed; MI: meconium ileus; NBS: newborn screening; PI: pancreatic insufficiency; PS: pancreatic sufficiency.

**Table 4:** Summary overview of the studies evaluating the association between breastfeeding and anthropometric outcomes in cystic fibrosis.

First Author (Year of Publication) [Reference]	Comparisons	Length/Height	Weight/BMI
Holliday et al. (1991) [20]	BF ≥ 3 m vs. no BF or BF< 3 m	No significant differences in Lz at the age of 3, 6, 12, 18 and 24 m among infants without MI. In the subgroup of infants with MI, mean values of Lz at the age of 24 m were higher among infants BF ≥ 3 m as compared to those not BF or BF < 3 m: Lz at 24 m: 0.94 vs. −0.85, *p* = 0.01.	No significant differences in Wz at the age of 3, 6, 12, 18 and 24 m among infants without MI. In the subgroup of infants with MI, mean values of Wz at the age of 12 and 24 m were higher among infants BF ≥ 3 m as compared to those not BF or BF < 3 m: Wz at 12 m: 0.96 vs. −1.19, *p* = 0.001, Wz at 24 m: 0.94 vs. −0.85, *p* = 0.007.
Parker EM et al. (2004) [13]	noBF, BF supplemented with formula <6 m, BF supplemented with formula ≥ 6 m, exBF <6 m, exBF ≥ 6 m	Not evaluated	No significant differences in BMI (*p* = 0.67)
Colombo et al. (2007) [14]	BF > 4 m vs. no BF or 1–4 m BF (any type of BF: exclusive, predominant, partial)	No significant differences in Lz (at the time of enrollment and at 1 year of age). Mean Lz values at 1 year of age were −0.84, −1.03 and −0.70 among no BF, BF 1–4 m and BF > 4 m infants, respectively (*p* = 0.47).	No significant differences in Wz (at the time of enrollment and at 1 year of age). Mean Wz values at 1 year of age were −0.51, −0.68 and −0.44 among no BF, BF no BF, BF 1–4 m and BF > 4 m infants, respectively (*p* = 0.54).
Jadin et al. (2011) [15]	exBF < 1 m, 1 m, ≥ 2 m and exclusive standard caloric density formula (exFF20, 20 kcal per ounce) and high caloric density formula (exFF22, 22 kcal per ounce)	No significant differences in Lz.	Rapid decline in Wz from birth to 6 m of age observed only in children with exBF > 2 m (*p* < 0.0001).
Leung et al. (2017) [17]	exBF at 3 m of age vs. exFF or combination	Not evaluated	Wz at 3 m of age higher among exBF infants as compared to exFF and those receiving a combination of FF and BF (mean difference: 0.54, 95% CI: 0.22 to 0.87). However, no differences emerged when exBF infants were compared with exFF infants (mean difference at 3 m of age: 0.13, 95% CI: −0.17 to 0.44). No significant differences at 6 and 12 m of age.
Munck et al. (2018) [18]	Different comparisons for nutritional outcomes (exBF vs. exFF during the first 3 m of life) and pulmonary outcomes (exclusively or partially BF for 3 m vs. exFF)	No significant association between exBF and Lz ≥ 10th at the age of 2 years (OR: 0.66, 95% CI: 0.22–1.96).	No significant association between exBF and Wz ≥ 10th percentile (OR: 0.39, 95% CI: 0.08–1.99).

BF: breastfed; BMI: body mass index; CF: cystic fibrosis; CI: confidence Intervals; exBF: exclusively breastfed; exFF: exclusively formula fed; exFF20: exclusive standard caloric density formula (20 kcal per ounce); exFF22: exclusive high caloric density formula (22 kcal per ounce); FF: formula fed; Lz: length-for-age z score; MI: meconium ileus; OR: odds ratio; Wz: weight-for-age z score.

**Table 5:** Summary overview of the studies evaluating the association between breastfeeding and pulmonary outcomes in cystic fibrosis.

First Author (Year of Publication) [Reference]	Comparisons	Respiratory Function	Lung Infections and Other Pulmonary Outcomes
Parker EM et al. (2004) [13]	noBF, BF supplemented with formula <6 m, BF supplemented with formula ≥ 6 m, exBF <6 m, exBF ≥ 6 m	No significant differences in FEV_1_% collected at the time of filling out the survey questionnaire (*p* = 0.42). FEV1 values were ≤70%, 71-90% and >90% in 20, 33 and 47% of patients who were not BF as compared to 16, 31 and 55% of patients who were exBF ≥ 6 m.	Percentages of patients who received 0–1, 2–3, ≥4 courses of IV antibiotics over the 2 years prior to the enrolment were 72, 16 and 12% among no BF and 84, 10 and 6% among those exBF ≥ 6 m (*p* = 0.03). No significant differences in age at onset of symptoms (*p* = 0.28): 64, 75, 82 and 90% of patients who were not BF had symptoms onset by 3, 6, 12 and 24 m, respectively, as compared to 60, 72, 79 and 87% among patients who were exBF ≥ 6 m.
Colombo et al. (2007) [14]	BF > 4 m vs. no BF or 1–4 m BF (any type of BF: exclusive, predominant, or partial)	Mean FEV_1_% values at the time of enrollment were 91, 98 and 112 among no BF, BF 1–4 m and BF > 4 m infants, respectively (*p* < 0.001). Mean FVC% values at the time of enrollment were 91, 98 and 111 among no BF, BF 1–4 m and BF > 4 m infants, respectively (*p* < 0.001).	Mean number of infections during the first 3 years of life was: 5 among patients with BF > 4 m, 7.5 among those with BF 1–4 m and 8 in the no BF group (*p* = 0.015). No significant differences in number of hospitalizations during the first 3 years of life.
Jadin et al. (2011) [15]	ExBF < 1 m, 1 m, ≥ 2 m and exclusive standard caloric density formula (exFF20, 20 kcal per ounce) and high caloric density formula (exFF22, 22 kcal per ounce)	Not evaluated	Percentage of never colonized with P. *aeruginosa* through the first 2 years of age higher among infants with exBF = 1 m (90%) as compared to exBF < 1 m (44%), exBF ≥ 2 m (40%), exFF20 (43%) and exFF22 (50%). Percentage of patients who had ≥ 2 P. *aeruginosa* infections through the first 2 years of age lower among exBF = 1 m (0) and exBF ≥ 2 m (0) as compared to BF < 1 m (13%), exFF20 (32%) and exFF22 (43%) (*p* = 0.026 for all group comparison and *p* = 0.003 for BF vs. all FF infants). Mean values of Wisconsin CXR scores at the age of 2 year lower among exBF = 1 m (2.0) as compared to exBF < 1 m (4.5), exBF ≥ 2 m (5.7), exFF20 (3.4) and exFF22 (4.1) (*p* = 0.015). No significant differences in S. *aureus* infections.
Rosenfeld et al. (2012) [16]	BF in infancy vs. no BF	Not evaluated	No significant association between BF and P. *aeruginosa* acquisition (HR: 0.85, 95% CI: 0.60–1.21)
Munck et al. (2018) [18]	Different comparisons for nutritional outcomes (exBF vs. exFF during the first 3 m of life) and pulmonary outcomes (exclusively or partially BF for 3 m vs. exFF)	Not evaluated	No significant differences in P. *aeruginosa* acquisition (37% among exFF infants and 23% among exclusively or partially BF infants, *p* = 0.10). Initial acquisition of P. *aeruginosa*> 1 year (25% among exFF infants and 64% among exclusively or partial BF infants, *p* = 0.06)
Fitzgerald et al. (2020) [21]	BF of variable duration vs. never BF	Not evaluated	No significant association between BF and hospitalization for pulmonary exacerbation in the first 36 m of life (OR adjusted for type of diagnosis, sex, sibling with CF, parent smoking, private health insurance and genotype: 1.85, 95% CI: 0.84–4.07.)
Padoan et al. (2019) [19]	BF of variable duration vs. never BF	Not evaluated	The risk of negative outcome as defined by chronic P. *aeruginosa* infection and/or faltering growth at 1 year of age was higher among never as compared to BF infants (OR 2.92, *p* = 0.061)

BF: breastfed; CF: cystic fibrosis; CI: confidence intervals; CXR: Chest X ray; exBF: exclusively breastfed; exFF: exclusively formula fed; exFF20: exclusive standard caloric density formula (20 kcal per ounce); exFF22: exclusive high caloric density formula (22 kcal per ounce); FEV1: forced expiratory volume in one second; FVC: forced vital capacity; NBS: newborn screening; OR: odds ratio.

## Data Availability

Not applicable.
